# Supplementary figures and images for: Detection and phylogenetic analysis of adenoviruses occurring in a single anole species
Source: PeerJ. 2018 Aug 29;6:e5521. doi: 10.7717/peerj.5521 (PMC6119460; doi:10.7717/peerj.5521)

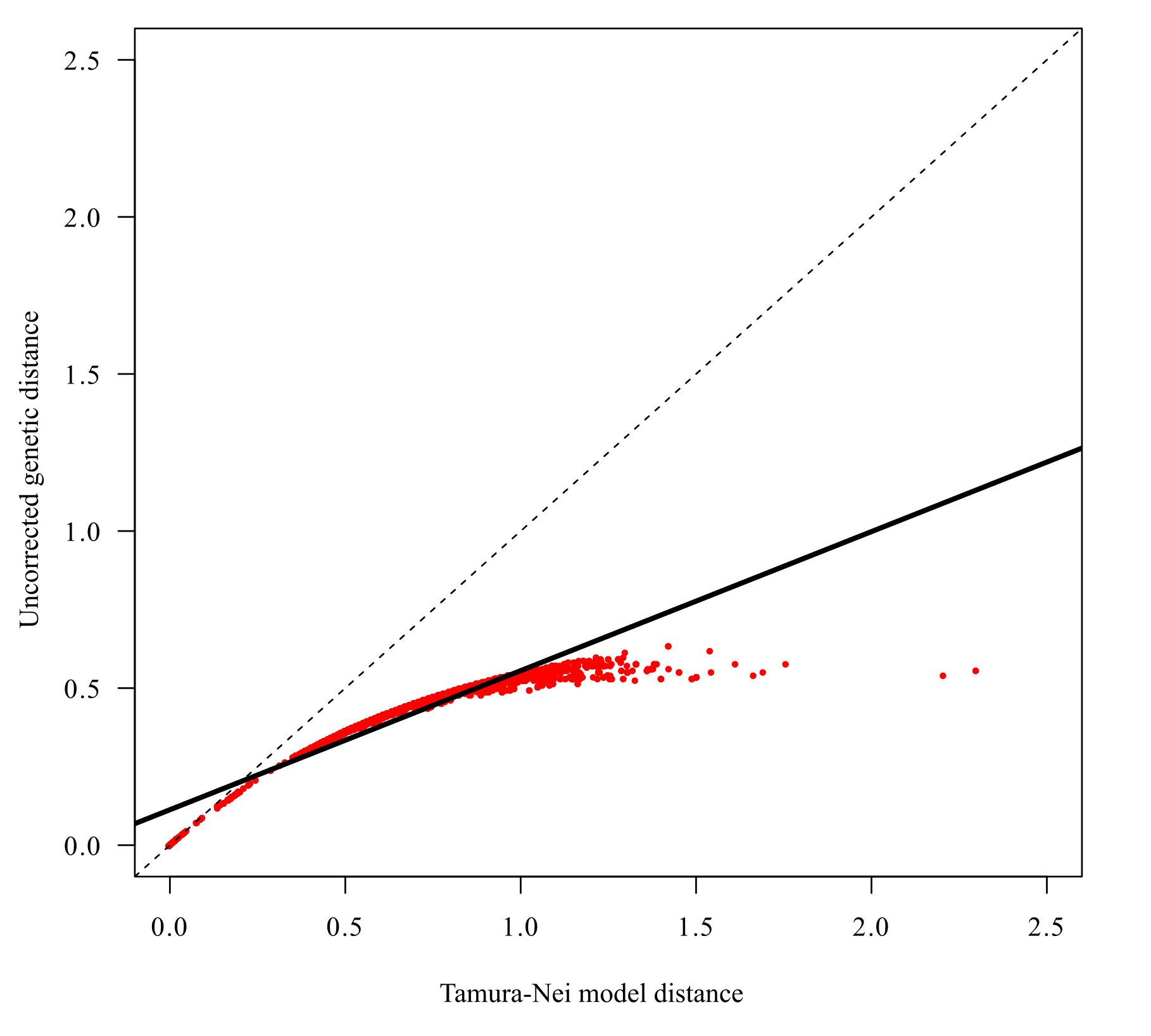

Supplement: Supplemental Information 3 — Model-corrected genetic distances (Tamura-Nei) are plotted against pairwise genetic distances. The dotted line represents a slope of 1. The solid line represents the linear regression of model-corrected distances against pairwise genetic distances. The plateau curvature of the cloud of points suggests that the nucleotide alignment is saturated. [file peerj-06-5521-s003.png]
